# An Alternative Correct Answer to the Cognitive Reflection Test

**DOI:** 10.3389/fpsyg.2021.662222

**Published:** 2021-08-23

**Authors:** Petra Filkuková, Johannes Langguth

**Affiliations:** ^1^Department of Psychology, Inland Norway University of Applied Sciences, Lillehammer, Norway; ^2^Simula Research Laboratory, Lysaker, Norway

**Keywords:** cognitive reflection test, alternative correct answer, text ambiguity, reflectivity, analytical reasoning

## Introduction

### The Cognitive Reflection Test

The Cognitive Reflection Test (CRT) measures one's tendency to engage in reflective deliberate System 2 thinking. The CRT consists of three mathematical tasks, which are designed to generate an intuitive wrong answer (Frederick, 2005). It requires cognitive reflection to override this intuitive answer (which is typically considered first) and come up with the correct answer. The CRT has quickly reached wide popularity and within 16 years has been cited by over 4,800 publications according to Google Scholar (~2,000 citations in Scopus). Studies which applied the CRT focused for instance on thinking biases (Stanovich and West, 2008), time and risk preferences (Oechssler et al., 2009), fluency (Alter et al., 2007), performance on heuristics-and-biases tasks (Toplak et al., 2011), and decision-making tasks (Campitelli and Labollita, 2010). High scores on the CRT correlate positively with numeracy, actively open-minded thinking and working memory and negatively with paranormal beliefs, denominator neglect and belief bias in syllogistic reasoning tasks (Toplak et al., 2011; Baron et al., 2015; Sirota and Juanchich, 2018). The original test also inspired the development of other tests of cognitive reflection, based on the same principle of “tricky” items, which evoke an intuitive wrong answer, either numerical or verbal (e.g., Toplak et al., 2014; Thomson and Oppenheimer, 2016).

The original test included open-ended questions, but studies investigating the possibility of presenting it as a multiple-choice task later emerged (Travers et al., 2016). Sirota and Juanchich (2018) proved that presenting participants with the correct answer in a multiple-choice format does not significantly increase the number of correct responses, even in situations when only two options are presented (the correct and the intuitive answer).

For ease of correcting, tasks designed for evaluating cognitive performance, including the CRT, typically include only one correct answer. In a school setting, the existence of potential alternative correct answers is often discovered in situations when stakes are high, for instance when a student scrutinizes every answer on the test in search of a missing point which s/he needs to pass an exam. However, in a research setting, participants who complete tasks measuring cognitive skills do not have any feedback on their performance as questionnaires are often anonymous and distributed online. Hence, in the vast majority of the data collections on the CRT, respondents do not receive feedback on their individual performance and thus also do not feel urged to explain the researcher their reasoning regarding an answer which was evaluated as wrong. In such a context the risk that a potential alternative correct answer on the CRT might stay undetected is higher than in the case of tasks in school exams.

There has generally been little attention to alternative wrong answers in the studies using the CRT, as they are less frequent than the intuitive wrong answers. When Frederick introduced the test, he reported that women reached a lower number of correct answers than men and additionally, for every CRT item, women were more likely to provide the intuitive answer, whereas men were more likely to give other types of wrong answers (Frederick, 2005, p. 37). Frederick concluded that even if men reply erroneously, they tend to reflect more on the answers and are less inclined to accept the intuitive answer, which suggests that he acknowledged that the non-intuitive wrong answers require higher levels of reflection than the intuitive wrong answers. Apart from the lower frequency of alternative answers in general and their relative higher frequency among men, Frederick did not elaborate on the alternative answers to the CRT. Alternative wrong answers are often presented as indistinguishable from the intuitive answers and both count for zero points in the total score of cognitive reflection. Although all types of wrong answers are inferior to the correct answers, one can argue that participants who provide intuitive answers are not identical with participants who provide other types of incorrect answers. Reasons for the other incorrect answers may differ greatly: participants might have either successfully rejected the intuitive answer but did not manage to find the correct answer, or participants did not even consider the intuitive answer and answered according to an entirely different line of reasoning.

## The Lake With Lily Pads: An Alternative Correct Answer

*In a lake, there is a patch of lily pads. Every day, the patch doubles in size. If it takes 48 days for the patch to cover the entire lake, how long would it take for the patch to cover half of the lake?* (intuitive answer: 24 days, correct answer: 47 days).

The lake scenario is the only one of the three CRT items which focuses on the topic of exponential growth. Perhaps even more than in the case of the other two CRT items where participants compute price of a ball and production duration in a factory, participants need to understand the hypothetical nature of the scenario (as from their own life experience they may know that not even the fastest growing water lily Victoria reaches such speed of growth). Regardless how artificial the lake example sounds, appreciating the speed of exponential growth has practical implications and is useful for instance for understanding the dynamic of the COVID-19 pandemic.

In the context of a study on evaluation of misinformation, we distributed the CRT in a paper version. When entering the data into a statistical program, only correct answers were recorded and no further attention was given to wrong answers. The situation has changed after one male participant elaborated on his reasoning for providing the answer “***1*** ***day***” for the lake-example. He noted that ***we did not specify which half of the lake*** ***we ask about***and wrote that ***the first half of the lake gets covered by lily pads in*** ***47 days, whereas the second half of the lake gets covered by lily pads in 1 day***. Hence, this participant reflected on the example even more than the participants who provided the correct answer. He successfully rejected the intuitive answer 24 days, but then he reflected also on the standard correct answer “47 days” and concluded that it may be a trick too, as there are different answers for the first and the second half of the lake. Nevertheless, following the standard way of dividing the answers into correct (reflective) and incorrect, this answer would end up in the same category as intuitive wrong answers and receive zero points in the score of cognitive reflection. This answer was not frequent in the total number of answers (given that most participants answered either the correct or the intuitive answer), yet it was not uncommon among the other types of answers. Before reading the explanation of this participant, we did not understand the reasoning behind this answer and we perceived the answer “1 day” as even more wrong than the other wrong answers which were numerically closer to the correct answer. We believe that all participants who answered that half of the lake would get covered by lily pads in 1 day most likely followed the same logic and reported the more striking speed of lily pads' growth, which concerned the second half of the lake. We find it highly unlikely that participants answering “1 day” could have the first half of the lake in mind, despite having the information that the entire lake gets covered by lily pads in 48 days and that the area covered by lily pads doubles in size every day, as this would imply that they concluded that it takes the remaining 47 days for the second half of the lake to get covered by lily pads. As the frequency of this alternative correct answer is not high among participants, it is unlikely that it significantly affects the results of most of the studies. However, we believe that it is not defendable to categorize an answer as incorrect only because it is not frequent. Similarly to the exam situation mentioned in the introduction, alternative correct answers are typically rare, as they did not come to mind to the author of the test/exam as well as to the majority of the participants. In the same way as it was not possible to foresee that the CRT would have an alternative correct answer, it is also impossible to know with certainty that categorizing the alternative correct answer as incorrect will never have any impact in case of such broadly used test (for instance when it comes to *p*-value reaching significance, or in case of job applicants screened by the CRT).

## Conclusion

We suggest counting the answer “1 day” on the lake scenario as correct, as it is complementary to the answer 47 days, only referring to the second half of the lake. Such addition would not anyhow discredit the widely used test of cognitive reflection and would only contribute to its scoring instructions.

Alternatively, it should be specified in the task which half of the lake is meant, as in its current form it is ambiguous and participants who report the (more striking) speed of coverage of the second half of the lake cannot be dismissed as incorrect. We believe that a person who answers that half of the lake is covered in 1 day solved the key element of this task, namely that it takes 1 day to cover the second half the lake.

A third option is to use the test only in multiple-choice response format. Participants who would in an open-ended question answered 1 day would in such situation most likely realize that the other half of the lake is meant than they had in mind and consequently choose the option 47 days.

Additionally, we propose that more attention should to given to the possibility of existence of alternative correct answers also in other performance tests used in psychological research, as a wide range of acceptable answers is typically assessed only in the case of projective tests.

## Author Contributions

PF and JL discussed the opinion presented in the paper. PF wrote the manuscript and JL approved it. Both authors contributed to the article and approved the submitted version.

## Conflict of Interest

The authors declare that the research was conducted in the absence of any commercial or financial relationships that could be construed as a potential conflict of interest.

## Publisher's Note

All claims expressed in this article are solely those of the authors and do not necessarily represent those of their affiliated organizations, or those of the publisher, the editors and the reviewers. Any product that may be evaluated in this article, or claim that may be made by its manufacturer, is not guaranteed or endorsed by the publisher.
